# Typologies of Extreme Longevity Myths

**DOI:** 10.1155/2010/423087

**Published:** 2011-03-15

**Authors:** Robert D. Young, Bertrand Desjardins, Kirsten McLaughlin, Michel Poulain, Thomas T. Perls

**Affiliations:** ^1^New England Centenarian Study, Geriatrics Division, Department of Medicine, Boston University Medical Center, Boston, MA 02493, USA; ^2^Département de Démographie, Université de Montréal, Montréal, QC, Canada H3C 3J7; ^3^FNRS, IACCHOS, Université Catholique de Louvain, 6000 Charleroi, Belgium

## Abstract

*Purpose*. Political, national, religious, and other motivations have led the media and even scientists to errantly accept extreme longevity claims prima facie. We describe various causes of false claims of extraordinary longevity. *Design and Methods*. American Social Security Death Index files for the period 1980–2009 were queried for individuals with birth and death dates yielding ages 110+ years of age. Frequency was compared to a list of age-validated supercentenarians maintained by the Gerontology Research Group who died during the same time period. Age claims of 110+ years and the age validation experiences of the authors facilitated a list of typologies of false age claims. *Results*. Invalid age claim rates increase with age from 65% at age 110-111 to 98% by age 115 to 100% for 120+ years. Eleven typologies of false claims were: Religious Authority Myth, Village Elder Myth, Fountain of Youth Myth (substance), Shangri-La Myth (geographic), Nationalist Pride, Spiritual Practice, Familial Longevity, Individual and/or Family Notoriety, Military Service, Administrative Entry Error, and Pension-Social Entitlement Fraud. *Conclusions*. Understanding various causes of false extreme age claims is important for placing current, past, and future extreme longevity claims in context and for providing a necessary level of skepticism.

## 1. Introduction

People have long been fascinated with claims to extreme longevity. Ancient Roman historians attempted to tally reports of extreme age in local villages. Medieval European alchemists kept tabs on reports of centenarians, possibly to find a “cure” for old age (the Fountain of Youth). Inexplicably, various historians and even “scientists” such as Roger Bacon accepted outlandish and wild reports of extreme age prima facie, without a critical examination or inquiry into whether the ages reported were true. It was not until the 18th century, with the advent of demographers such as Georges Buffon (1707–1788) that a limit to the human life span was proposed, with Buffon stating that “the man who does not die of incidental diseases reaches everywhere the age of ninety or one hundred years" [1].

The first reasonable attempts at age validation were performed by demographers such as Adolphe Quetelet, who conducted a systematic investigation of purported centenarian ages appearing in the first Belgian census of 1846 [2]. In the 1870s, Sir William Thoms (who coined the term “folklore” in 1846 and subsequently investigated folk tales of extreme old age) suggested the need to question extreme ages claimed in folk tales. Thoms investigated extreme age reports provided by village elders in the context of old age data provided by life insurance companies [3]. In his time, no age greater than 103 years old (Jacob Luning in 1870) had been verified using insurance company records, far younger than the claimed ages that were well beyond 110 years. Despite this important lesson of considering context, 140 years later many people in the media and elsewhere are willing to accept a claim of 130 years despite the fact that the maximum proven age having been reached by a human is 122 years [4]. 

To provide a current context to unsubstantiated age claims, we provide here some statistics concerning supercentenarian (a person age 110 years or older) prevalence. Kestenbaum and Ferguson at the U.S. Social Security Administration reported Medicare data indicating that, in 2000, there were 32,920 centenarians and out of these, 105 or 0.3% were 110 years old and older [5]. Of 2,700 people who reportedly reached the age of 110+ years between 1980 and 1999, according to the SSA, only 355 (13%) could be confirmed. The US census listed 1,388 supercentenarians in 2000 (about 1 per 200,000) [6]. However, according to author R.D Young, per the surveillance efforts of the International Database on Longevity (IDL, http://www.supercentenarians.org/) and Gerontology Research Group (GRG, http://www.grg.org/), the number of living supercentenarians at present in the USA is approximately 60 to 70 (or approximately one living supercentenarian per five million people in developed countries and far fewer in less developed countries) and 250 to 300 world wide. 

Academics and lay people interested in age validation generally fall into two camps: the skeptics and the optimists. The initial skeptics were actuaries, who found that humans did not live beyond 113 years or so. Thomas Emley Young of the Institute of Actuaries, London, for example, attempted the first validated list of centenarians in the 1890s, finding no one older than 106 [7]. An initial acceptance of the claim of Pierre Joubert to be 113, by the Tache investigation in Canada in 1878, was later overturned [8]. Interestingly, Alexander Graham Bell purportedly attempted such a list in1918 [9]. 

Optimists, on the other hand, have tended to accept extreme age claims, prima facie, and provided rationalizations as to why these people were “healthy” and lived longer than the rest. For example, scientists such as Elie Metchnikoff, the inventor of the term “gerontology” circa 1903, tended to believe extreme age claims of 140 and above [10, 11]. Jean Finot, a transhumanist, believed, at the turn of the twentieth century, that the growing number of centenarians at the time and the improvement in average life expectancy portended the likelihood of human life spans of 150 plus years [12]. 1973 articles in *Scientific American* [13] and the *National Geographic* [14] reported people over the age of 120 years in the Russian Caucusus, and in Vilcabamba, Ecuador. But later its author, Alexander Leaf, became wary of these claims due to inconsistencies in the stories, and he engaged Richard Mazess and Sylvia Forman to further investigate the Vilcabamba claims, which were eventually found to be false [15]. Optimists paved the way for amazing unquestioned claims in the United States as well. Sylvester Magee was said to be aged “130” [16] and an “ex-slave” and Charlie Smith, who also claimed to be an “ex-slave”, was said to be age “137” when he died in 1979. Smith was later noted to be 100 years old at death based upon the 1900 census and was not, in fact, an ex-slave, having been born more than 15 years after the Emancipation Proclamation [17]. 

In 1955, given continued unbelievable extreme age claims, Norris and Ross McWhirter, the editors of the Guinness Book of World Records, noted the need to validate with sufficient records the “world's oldest person.” In 1986, Norris stated: “No single subject is more obscured by vanity, deceit, falsehood and deliberate fraud than the extremes of human longevity” [18]. 

A resurgence of longevity myths in the 1970s, particularly in the Caucasus region of Soviet Russia, the Hunza Valley in Pakistan, and the Vilcabamba valley in Ecuador was finally debunked by objective scientific investigation in the early 1980s [15, 19, 20]. Even the skeptical Guinness Book of World Records was not infallible, however. For example, Shigechiyo Izumi of Japan was accepted as aged 113 years in 1978 and was thought to be the oldest verified person ever at age 120 years in 1986. However, in 1987 he was determined by Japanese researchers to more likely be only 105 years old at the time of his death [21]. Unfortunately in 2009, as discussed below, the fantastic age claim of Sakhan Dosova of Kazakhstan, age “130 years” (1879–2009), was supported in an issue of *Scientific American* [22] despite the lack of early-life documentation. Also in 2009, there was the claim of Tuti Yusupova of Uzbekistan who was claimed to have been born on July 1, 1880 and therefore was alleged to be “129” in 2009. The BBC news reported the event of her birthday as if it were valid, noting a “birth certificate.” However, even the report's own video clip shows that the document was a late-life one issued in 1997, not proof of birth issued in 1880, or anywhere close to it [23]. Surprisingly, these and other similar reports provide little in the way of skepticism even when the individuals were not claimed as the oldest ever, seven or eight years earlier, when they would have broken the accepted record of 122 years, 164 days set by Jeanne Calment of France (February 21, 1875–August 4, 1997) [4]. Many claims, such as the one appearing in *Scientific American*, are characterized by geographically specific absences of records from the late 1800s and early 1900s from regions such as Armenia where most records were destroyed by the war. In the case of China, ages are traditionally recorded in 12-year increments or animal cycles according to the Chinese zodiac, and therefore animal signs rather than birth years are often more culturally salient among the oldest old. Additionally, in China one can encounter the tradition of ages beginning at the number one, rather than zero, which can lead to an additional year, as in the case of a former first lady of China, Madame Chiang Kai-shek, who died at age of 105, not 106 [24]. 

Despite this history of the overwhelming improbability of various extreme age claims, the Western media continue to report such claims, particularly from exotic regions, as if they might be true. Meanwhile, as discussed below, well-documented and validated cases generally do not exceed 115 years of age. The record for Germany is just 112 [25]; for Sweden, 113 [26]; for Italy [26] and Spain [27], 114. Since 1837, with the advent of compulsory birth registration, no one in the UK has been proven to survive beyond the age of 115 years [26]. Where birth registrations are available in the mid to late 19th century, valid claims of ages beyond 122 do not exist. As a result, in our experience, claims to age 130 exist only where records do not. 

The problem, however, extends beyond the media. In 2007, Professor Orhan Kural, of Turkey, supported the dubious claim of Seher Bulut, age “122” [28, 29], despite no proof of birth and a generation gap suggesting that this woman gave birth at an age reasonably beyond menopause. Government officials have been willing to provide a benefit of the doubt in some cases, perhaps because of political pressure or community notoriety rather than any sincere desire to seek the truth. Even in the USA, the claim of William Coates to be “114” was incorrectly accepted by Dr. Irving Smith of the Evelyn Cole Senior Center, Maryland, in 2004. Census research subsequently showed that Mr. Coates was only 92, not 114 as claimed [30, 31]. 

More scientifically rigorous treatment of the subject came about in the late 1980s and early 1990s; a group of demographers and gerontologists came together in a series of workshops to formulate criteria for effectively validating or invalidating extreme age claims. These efforts led to the International Database on Longevity (IDL, http://www.demogr.mpg.de/en/research/695.htm and http://www.supercentenarians.org/), an ongoing list of validated supercentenarians that is well described in a recent monograph produced by the Max Planck Institute for Demographic Research [32]. A number of monographs have been written by these experts on the subjects of age validation and invalidation [2, 32–34]. Another group, based in Southern California, named the Gerontology Research Group (GRG, http://grg.org/) and led by L. Stephen Coles, was formed to facilitate, particularly via the internet, a group of academic and lay investigators interacting with one another in the maintenance of a validated claims list that was begun in 1999. This effort eventually branched into an actual records database of supercentenarian cases. Finally, the ongoing efforts of the Guinness Book of Records also facilitate the adjudication of world record age claims, providing a “final appeals” process whereby any claim from around the world may be submitted. This was the case in 2005, when the claim of Maria Capovilla of Ecuador, said to be 116, was submitted [35]. The documents were deemed sufficient by Guinness research, and subsequent follow-up research by other groups, such as the International Database on Longevity, tended to agree with this conclusion. Of note, Ms. Capovilla lived in a big city near sea level (Guayaquil) and her age did nothing to bolster the Vilcabamba myth that people living high in the Andean mountains, far from big cities, had an extreme survival advantage. 

A number of ongoing studies of human exceptional longevity, for example, the Georgia Centenarian Study [36, 37], the Ashkenazi Jewish Centenarian Study [38], the Okinawa Centenarian Study [39, 40], the Long Life Family Study [41], and the New England Centenarian Study [42, 43] have relied upon the age validation criteria formulated by the IDL in making sure that the claimed ages of their subjects are real. The results of all these efforts are in remarkable agreement that verified age claims above 115 are extremely rare.

The New England Centenarian Study (NECS) has, over the past five years, made a concerted effort to specifically enroll supercentenarians (age 110+ years). Because supercentenarians are so rare at approximately one per five million people in the United States, the NECS recruits and enrolls these subjects from throughout North America. The study has, to date, the largest such sample in the world with over 100 subjects attaining ages of 110–119 years [44]. The NECS recruitment and enrollment experience, along with Robert Young's broader experience, since 2000, of monitoring and validating or disproving supercentenarian claims for Guinness World Records [45] and since 1999 for the GRG, has led to our ability to observe and categorize some of the different reasons and causes of inaccurate claims of extraordinary ages. It is important for researchers studying supercentenarians to be aware of signs that the age being claimed may be false. Identifying typologies of invalid age claims that we and others [1, 46, 47] have encountered provides a contextual background to the striking age claims often reported in today's media, while knowledge of the demographics of supercentenarians helps us to place extreme age claims of 110+ years in proper context. Thus, our purpose here is to classify the various causes or reasons for false age claims, while providing a backdrop that places these claims in proper demographic context. A more general knowledge of the typical circumstances or motivating factors that underlie age misreporting may be helpful in decreasing irresponsible coverage and inclusion of such claims in government records and scientific research.

## 2. Methods

To obtain an estimate of how the rate of invalid age claims in an American sample changes with age, one of the authors, RY, queried Social Security Death Index (SSDI) data which cover about 95% of the deaths in the USA in a given year to determine the number of people with birth and death years that yielded ages greater than or equal to 110 years (e.g., someone listed as born in 1870 and dying in 1981 would have been 110 or 111; someone born in 1870 and dying in 1982 would have been 111 or 112). A list was then generated for possible deceased supercentenarians from 1980 to 2009 according to age (column 1 of Table 1). A range including two possible ages is listed for each row because the months and days of birth and of death were not included in the age calculation from the SSDI data (to do so would have made the review of the SSDI data too arduous and our priority in looking for SSDI-generated cases of supercentenarians was sensitivity, not specificity). Without the day and month data, the person's age of death could have been, for example, 110 or 111 years. This is why the age of 109 years is also included in the table, even though 109 years would not qualify the person as a supercentenarian. This list was then compared to the results of the ongoing validation effort conducted by Robert Young and colleagues associated with the Gerontology Research Group (GRG) for supercentenarians in the USA who died during the same time period (column 2). The GRG had access to name, exact dates of birth and death, and in most cases vital information about the potential supercentenarian's parents and siblings for all of the SSDI-generated cases. Other purported supercentenarians were located by the GRG via surveillance of the lay press and regular searches of the internet as well as referrals to the GRG by friends or family of the individual. A comparison of the two columns then yielded a valid claims rate. For example, the SSDI listed 24 persons born in 1870 and dying in 1980, for a total of 24 potential 110-year olds. Of these, four were listed as verified (about 17%). 

In the course of validating cases for the NECS, GRG, and Guinness World Records, Robert Young has used the set of rules described below and summarized in Table 1. Generally, the rate of validation for potential oldest subjects in the NECS is high because these individuals are usually around ages of 107–110 years old, nearly all of them come from the USA and Canada (as noted in the results, higher ages and claims from less developed countries have lower validation rates), and their reason for cooperating with the NECS in the age validation process is to volunteer for study on the genetics of exceptional longevity. Cases adjudicated for Guinness tend to have much higher invalid rates (and therefore there is an ascertainment bias) because the claimed ages are much higher (approaching or surpassing the oldest age record). The validity rates for the GRG tend to be somewhere in between the NECS and Guinness because ages for the GRG list begin at 110 years, but the list also includes claims from all over the world with some coming from less developed countries or regions. Still, the vast majority of GRG cases come from the United States, Western Europe, Japan, Australia, and Canada. The ascertainment bias that is likely associated with the GRG effort enhances the sensitivity of the above validation effort for the oldest ages and strengthens the case that nearly all the claimed ages, particularly those in the United States, come to the GRG's attention and are therefore not missed.

Authors B. D. and M. P. also have a vast experience in assessing the validity of cases. It is out of the collective experience of the authors, as well as from a literature and media review, that a list of typologies for invalid cases was constructed.

The ability to validate an age claim is dependent upon the various resources available for that claim, and these in turn can vary according to the administrative, cultural, or religious settings. These data can increase the probability of either a true positive or a true negative age claim. Ideally, the person's original birth record or a certified copy of the record, registered at, or shortly after, the person's actual birth should be available. Importantly, one must be certain that the record relates to the person under scrutiny, namely, on the basis of first and last name, place of birth, and the parents' names, which are the elements that are normally available on the birth record. In the cases of the most extreme old, by virtue of their rarity and likelihood of being false, the birth record alone should not be sufficient because of the possibility, for example, of homonyms or namesakes (a person named after another person). 

Consistency between birth, death, and marriage records according to name, surname, and parent and spouse names incrementally increases the probability that the alleged age is true. The ideal validation procedure must thus include a “family reconstitution,” that is, an identification of the timing and composition of the births of the entire sibship in relation to the parents and their birth data to insure that there is no possibility of mistaking identity and to be sure that the birthdates make sense in relation to one another [33]. The use of family pedigree reconstitution and a check of other vital records, such as court documents, may be employed if the age claim is extreme (such as that of Jeanne Calment) [4]. 

A chapter by Michel Poulain appearing in a recent monograph on supercentenarians [34] provides a detailed discussion of the necessary steps for validating extreme age claims. As discussed in this contribution, all necessary investigations have to be consistent in order to prove validity of age. If one important piece of information is missing, the age cannot be validated with “no doubt at all.” Nevertheless the lack of an important piece of information (e.g., no birth record) does not necessarily invalidate the claim, but rather precipitates the need for additional data to support the age validation. A proxy or substitute record, such as the 1900 census for someone born in 1897, listing them as age 3 for example, may be counted as a sufficient replacement for proof of birth. Internationally, the proxy-birth rule is limited to documents issued within 20 years of the birth event [4]. On the other hand, a single piece of information (such as the age of the oldest child) may make the claimed age highly improbable. For example, when Antisa Khvichava recently celebrated her “130th” birthday [48], the age of her oldest son (70) placed the claim squarely in the “probable age exaggeration” category: her age report is likely off by 20–40 years. If one element is wrong, the entire validation process will be considered as highly improbable, and it is definitively easier to prove that this person is not a centenarian than the opposite. In fact, the validation will never be final, while the invalidation is generally final when clear grounds for invalidation are found. 

The principles of age validation that we rely upon are summarized below

**Basic Principles for Age Validation**

- Ideally, age should be calculated from linking a birth registration with a death registration or with a living person.
- In the absence of a birth registration, date of birth should be obtained from a document dating back as far as possible, and specifically be dated close to the person's alleged birth (e.g., a local census record).
- The identification of the person must be unequivocal, thus necessitating matches with the names of parents and siblings or spouses and children, and place of birth.
- Family reconstitution.
- Independent corroboration is required when name identification is not sufficient, in the form of other records recording age which do not rely on the same base source.
- Life events-marriage, birth of children, schooling-must be consistent with the alleged date of birth.
- The requirements of age validation should be proportionate to the exceptionality of the age claim. For example, claiming to be age 120 will require many different and consistent forms of proof (as was done with Jeanne Calment), while claiming to be 110 years old requires only the three basic proofs: proof of birth, survival to age 110 (identification), and proof that the person in the birth record is the person in the ID record. It has been suggested that for ages 105–109, proof of birth and death should be sufficient.

## 3. Results

In the comparison of supercentenarian cases generated by the Social Security Death Index to a validated list generated by the GRG, one observes that the rate of validation declines with age, from about 35% at age 110-111 to just 2% by age 115 and 0% for 120+ years (Table 1).

We provide below a list of eleven categories of how false claims emerge. Some categories are more historical while others are common causes of currently professed claims that are either proven false or do not have enough substantiating evidence to be believable. These categories are Religious, Patriarchal Myth, the Village Elder Myth, Fountain of Youth Myth (substance), Shangri-La Myth (geographic), Nationalist Pride, Spiritual Practice, Familial Longevity, Individual and/or Family Notoriety, Military Service, Administrative Error, and Pension Fraud. Each of these is elaborated upon below.

### 3.1. Religious, Patriarchal, Genealogical Myth

In tribal and village kinship networks, the eldest members can not only be a repository of wisdom and tribal knowledge, they can also be patriarchal or matriarchal figures and a living symbol of the family tree that holds together the past and present members of the family. Since very old age conferred status, some extreme ages were attributed to historically important individuals. Thus, in the Bible, Abraham is said to have lived “175” years. Some scholars believe that Aaron's age of “123” was meant to show that the priesthood was older than the law, represented by Moses (who lived to “120”) [49]. 

Moses's successor, Joshua, was said to have died at a younger age of 110 perhaps because he was of a lesser status than Moses, but still important and therefore older than the “average” age of 70–80 years stated in Psalm 90:10. With the more recent King David portion of the Bible, incredible ages give way to ordinary ages of death: 70 for David, or 58 for Rehoboam, for example.

In some cases, ages were exaggerated to extend a pseudo-genealogy further back into the past. For example, in ancient Sumeria, claimed ages corresponded to calendar cycles and special dates [50]. A later and reduced form of the cyclical-calendar genealogy myth was used in Japan, which inflated ages of emperors in an attempt to date Japanese history back to 660 BC [51]. This type of myth may no longer be a problem for current claims under investigation, but it is important when claims of ancient kings to be 117 or older are made. For example, Emperor Yao of China is said to have ruled for 100 years from age 17 to 117 (circa 2333 to 2234 BC) [52].

### 3.2. Village Elder Longevity Myth

The “village elder” myth can be considered as a localized version of the patriarchal myth; only it involves common people, not elite members of society. It is generally assumed that persons today cannot attain the ages of the ancients, but still one's “village elder” should be honored. The village elder myth originally centered around a tribal chieftain, but in places where local power was decentralized, elderly men and women began to lose such positions of power. Instead, the “village elder” became a source of pride, oral history, and a person to commemorate. The ages claimed tend to be limited by the masses' ability to believe them. Most claims of this type have been to ages less than 200 years old, with ages of 120 to 160 years seemingly representing the cusp of believability for the uneducated. The purported ages are commonly rounded off to the nearest five or ten years (called “age heaping”) (i.e., 125 or 130, not 123 or 129). These myths continue today in places such as Bangladesh, Nigeria, Indonesia, and Pakistan. 

**Figure 1:**
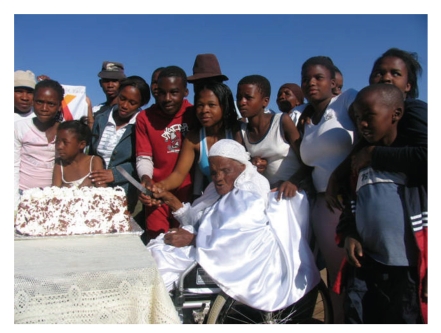
Moloko Temo of South Africa at what was announced as her 134th birthday. Photo with permission from the Department of Health and Social Development, Limpopo Provincial Government, South Africa.

A typical example of a “village elder” longevity myth is that of Moloko Temo of South Africa (Figure 1) [53]. Her identity card was issued in 1988, purporting that she was born on July 4, 1874. She died on June 3, 2009 supposedly at the age of “134 years.” There are no other documents substantiating her age. Most importantly, no one came forward with her age claim in 1988 when she would have become the oldest person in the world. The age of her children (some in their 70s) suggests that this woman was closer to 104 than 134. Interestingly, in interviews Temo discounted that she is the world's oldest person: “I think that there are others older somewhere” [54]. In other words, the motivation for her age might not be about national or international fame but rather local respect and a cultural reverence for extreme old age as exemplified by the local saying, “May you grow as old as the mountain, Khulu” (source: http://www.iol.co.za/news/south-africa/may-you-grow-as-old-as-the-mountains-khulu-1.217466#). 

It should be noted that the “village elder” myth is now often co-opted into a “nationalist” myth of longevity. The difference, however, is motivation; the original motivation for the village elder myth is local pride and joy; however the claim can then be discovered by the national press, and it becomes a source of national pride.

Another example is Ruby Muhammad, a woman who, as the “mother of the Nation of Islam” is said to be 113, but is more likely 103, according to the 1910 U.S. census photo with permission from the Department of Health and Social Development, Limpopo Provincial Government, South Africa. No birth certificate exists [55]. Her position is one of “matriarchal” status, a position of honor, and few details of her early life exist. Moreover, she was said to be illiterate before 1946, and misreported age is highly correlated with illiteracy.

### 3.3. Fountain of Youth Myth

Unlike the previously mentioned myths, which are rooted in patriarchal, ancient, and communal beliefs, the Fountain of Youth myth is anchored in the individual. The idea that people could change their environment (such as in alchemists' attempts to turn lead into gold), while not often supported by facts, became popular during the 1400s and 1500s. Consequently, Spanish conquistadors, already searching for fabulous cities of gold (the “Seven Cities of Cibola”), added the idea of finding the “Fountain of Youth.” Ponce de Leon explored Florida in 1513, seeking the fountain in vain.

The Fountain of Youth myth is connected to longevity in the idea of exampleism (or the “testimonial fallacy”). People need an example of success to believe that a special kind of water (e.g., “glacial milk” from the Andes), drug, or potion carries beneficial (magical) properties, bestowing extraordinary longevity on those who use it. To satiate this need, today's charlatans often provide made-up testimonials or anecdotes as “examples” of success (testimonial fallacy). The many websites and advertisements professing the age-reversing effects of growth hormone and other substances alleged to extend human longevity are a particularly egregious example due to the high frequency of adverse effects from these drugs and evidence that growth hormone actually shortens life span in adults [56, 57]. As an example, Dr. Norman Walker promoted “raw juicing,” and ages attributed to him were often 118, 119, 120, or even 130. Yet recent investigations found that he was only 99 years old [58].

### 3.4. Shangri-La Longevity Myth

An extension and adaptation of the Fountain of Youth myth is the idea that a particular place, rather than a substance, possesses what is needed to attain extreme age. Shangri-La was a fictional paradise in the 1933 novel *Lost Horizon. *Author James Hilton describes a place where the residents are happy, isolated, and live many years beyond the normal lifespan. This myth was particularly popular in the 19th century during the “Age of Empire” when people went in search of exotic and mystical lands (an adventure for wealthy Europeans, called the “Grand Tour”). Once again, we see wealth and personal vanity as motivating factors in longevity myths.

This myth differs from the Fountain of Youth myth in that it focuses on an entire village or mountain region, where the water, air, and so forth, are said to be qualitatively different than elsewhere. Modern examples of this myth include the Caucasus mountain region, the mountainous Vilcabamba region in Ecuador, and the Hunza Valley in Pakistan. In this type of myths, many people are claimed to achieve extreme old age. Thus, the Caucasus did not merely claim to have 168-year olds, but to have hundreds of people older than 120 years [59, 60]. In some cases, apparent age heaping showed how unreliable the claims were. Claimants were also disproportionately male, further incriminating the claim because the vast majority of centenarians are female.

**The Vilcabamba Claim**

This “Valley of Longevity” was promoted in the 1970s. Out of a total population of 819, the town boasted seven men and two women older than 100 years old. One man, Miguel Carpio, said that he was 123 years old. Another, Jose David, claimed to be 142 years old. Gabriel Erazo claimed to be 132 years old. Victor Maza claimed to be 120. The source of longevity was variously described as a pristine environment (“mountain water”), healthy habits such as constant movement, and isolation from the mainstream world. However, an investigation in 1979 by Dr. Richard Mazess of the University of Wisconsin, Madison and Dr. Sylvia Forman of the University of California, Berkeley found that there was not a single centenarian living in Vilcabamba [15]. The oldest person in the village was found to be 96. The average age of those claiming to be over 100 years was actually 86 years. Far from being a Shangri-La of very old people, the researchers concluded that: “Individual longevity in Vilcabamba is little, if any, different from that found throughout the rest of the world.”

Note that in the case of Vilcabama, there is overlap with other categories of age misreporting. Vilcabamba means “Sacred Valley” in the Inca language, thus invoking an association with religious and mythical beliefs. Also, nearly all the extreme age claimants were male, suggesting an overlap with the patriarchal and village elder myths. Usually, about eighty-five percent of centenarians [61] and ninety percent of supercentenarians are women [46].

**The Current Guangxi, China Claim**

**Figure 2:**
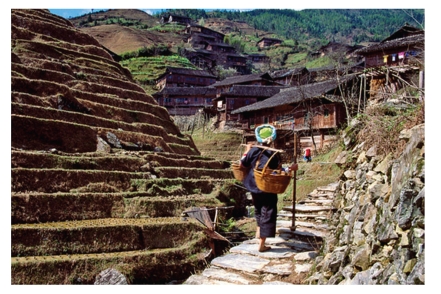
A village in Guangxi county, China (Permission, ChinaSpan.com).

This Southern region of China, which borders Vietnam, claims a “longevity cluster” with 74 centenarians, including a 113-year old, in a population of 250,000 (thus, more than twice the prevalence of centenarians in industrialized countries). Perhaps due to skeptical pressure, ages above 113 are not used here; instead the claim is focused on prevalence of centenarians, rather than maximum ages. Hotels and other entrepreneurs call the region a spa vacation destination, where, according to an October 11, 2008 Wall Street Journal article by Stan Sesser, “simply breathing the air, drinking the local water and eating meals there” is claimed to lead to better health and longevity (Figure 2) [62].

### 3.5. Nationalist Longevity Myth

An extension of the Shangri-La myth is the “Nationalist” longevity myth. The idea of the Nationalist longevity myth was rooted in the rise of Nationalism in the 19th and 20th centuries. As people's ideas became focused on their “one nation” versus another (with their nation being the “right” one, “powerful” one, “God-blessed” one, etc.), extreme age claims became a source of pride. 

**Figure 3:**
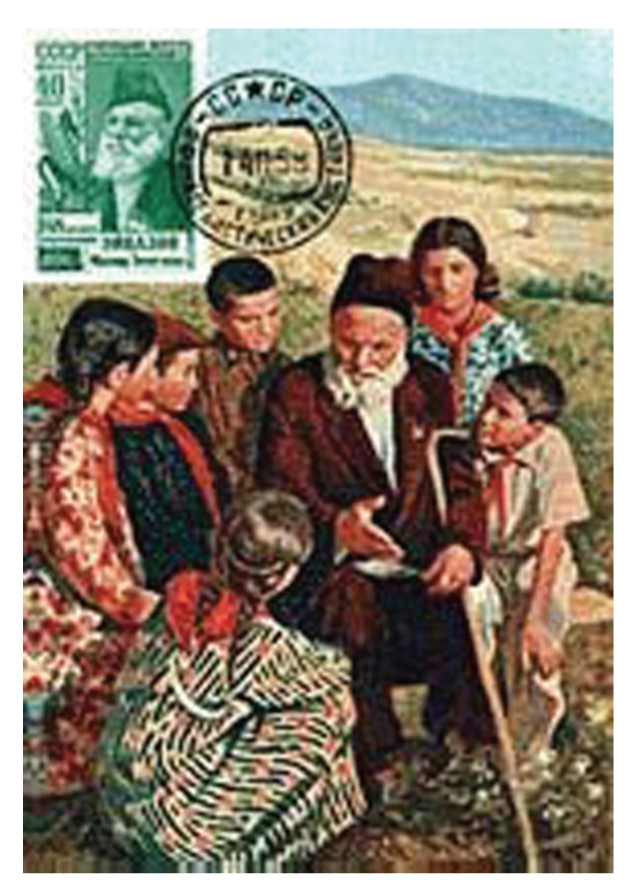
1956 USSR stamp depicting 148 years old Mahmud Eyvazov, the oldest person in the Soviet Union and living in Azerbaijan. The fact that a stamp was produced commemorating this man indicates the role of national pride in claims such as these (Permission, Azerbaijan International).

The Soviets used the longevity claims of the Caucasus to help promote the asserted superiority of the Communist way of life and their nation [60]. These claims seemed to spark a longevity contest between the USSR and USA. The USSR proclaimed that Shirali Mislimov of Azerbaijan was 168 years old when he died on September 2, 1973 (note that no Western journalist was permitted to interview “old Shirali”) [63]. Mahud Eyvazov was commemorated in a 1956 USSR stamp, for his 148th birthday and his being the oldest person in the Soviet Union (Figure 3) [63].

**Figure 4:**
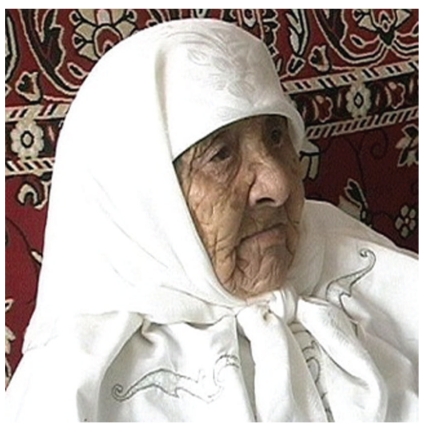
Sakhan Dosova's family and Kazakhstan officials claimed, as of April 1, 2009 that she was 130 years old. She died a month later. Photo permission from Radio Free Europe/Radio Liberty.

While the Soviet and American longevity race has lost its steam with the end of the cold war, extreme longevity claims from the former USSR still regularly appear in the press. Most recently, for example, Kazakhstan officials and the family of Sakhan Dosova claimed that she was the oldest person in the world at age 130 years old (Figure 4) (http://www.cbsnews.com/8031-504763_162-20010012-10391704.html) [22]. Perhaps this was a case of one-upsmanship since the claim appeared shortly after the Uzbekistan claim that Tuti Yusupova was 128. Most noteworthy about the Dosova claim is the fact that 8 years passed since she would have surpassed the long-held, accepted record of 122 years, and yet we only hear of the claim in 2009. Not surprisingly, there is no birth certificate supporting the claim; only a passport, a later-life census record, and an identification card, all of which could have been based upon one or the other. There are several other reasons why this claim is entirely unacceptable, including (1) the region from which the claim originates is well known for invalid claims with its poor recordkeeping, low rates of literacy, and a tradition of age inflation; (2) the claimed age is far beyond the accepted record holder and 16 years beyond the current oldest person in the world; (3) the ages of her children indicate that this claim is exaggerated, otherwise she would have given birth in her 60s [64]. 

Extreme age claims from Cuba, often in the 120–126 age range, have continued this tradition. Cuba even has a “120 club” [65], of which Fidel Castro is a member, as they wish him “120” years of health. The most recent heralded claim is a person they claim is the oldest person in the world, Candelaria "Candulia" Rodríguez, age 125 years [65]. The club relies upon a church register, indicating a birthdate of February 2, 1885, as proof. For such an extreme and potentially sensational claim though, multiple forms of corroborating proof are necessary (see text box above, providing principles of age validation) [66].

### 3.6. Spiritual Practice

This myth asserts that certain philosophies or religious practices allow a person to live to extreme old age. These types of myths are most common in the Far East. For example, some Daoists have claimed to live to over 200 years. In China, Li-Ching-Yuen was noted to be 256 years old when he died in 1933 [67]. Not only was his age claim fantastical, and the number chosen as a multiple of eight (considered good luck in China), but the rationale was that he lived so long due to his following a certain practice or way of life. This type of myth is also found in Buddhism. For example, Nyala Rinpoche claimed to be 142 in 1978 and to have attained a state where he no longer consisted of flesh but was “pure light” [68]. 

Hindu yogis often also claim extreme age, such as the Swami Bua, variously said to be “118” (http://www.yogasutranyc.com/pdf/2007-Bua-Flier.pdf) or even “120,” or Swami Kalyan Dev, who was noted to be “130 years old” (http://www.mangalyoga.com/team.htm). In a case such as this, followers can hope to live as long as the “Master” if they follow his guidance and direction. In the case of the spiritual practice myth, extreme age is associated with the supernatural and is often achieved through some activity. This is different than the concept of “religious blessing” common to monotheistic religions, whereby longevity is attained by finding grace or favor from God or gods, for example, the Religious Authority myth. Claims of this nature continue today.

### 3.7. Myths of Family Longevity

A relative living to an extreme age can be a source of significant pride for a family, and this is one of the most commonly encountered causes of inaccurate claims that we encounter. Many families relay stories of family members from many generations ago who lived to very old age. Often these ages are inflated, and there is no documented evidence for the claim. The farther back in time one goes, the easier it is to insert such a family member into the family tree. Sometimes one myth is used to prop up another. For example, Mattie Owens was claimed to be 119 years old in 2003 [69], and her son was said to be 87. An investigation by R.D. Young determined that Mattie was in fact 105 years old, and her son was just 80 years old [70]. These myths are quite common, even in the developed world. Macy Bare of North Carolina, said to be 115, turned out to be 107 [70]. In 2004, unequivocal census research revealed that William Coates of Maryland was 92, not 114 [30, 71].

The myth of persistent and extreme familial longevity is one of the more common typologies of age misreporting that we encounter, including countries such as the USA Though in the USA, it is relatively easy to find records, for example, from the U.S. census, to disprove claims, we encountered the claim of a Dominican Republic woman living in the U.S. who was supposedly 104 years old according to her immigration papers but her family indicated that she was “really 109” and that her mother lived to be 119, and her grandmother, to be 124 [72]. In reality, we do not even have sufficient proof that Ana Henriquez is 104, since the document was issued in 1963. Note that the age claims go higher the further back in the past the family tree goes. While many families insist a relative lived to “113” or even older, few families ever bother to investigate, and when they do, they are often disappointed.

### 3.8. Claims of Being the Oldest Person in the World: Individual and Family Notoriety

**Figure 5:**
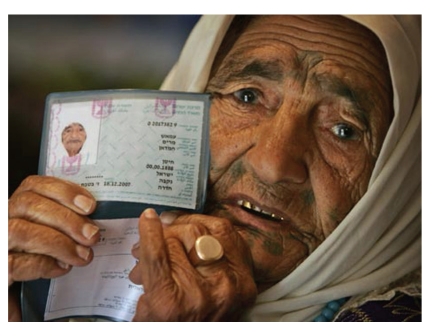
Mariam Amash's family states that “She rises every morning around five for prayers…. She then goes for a walk and then spends most of her day with the family. She recognizes all of us.” Such high function amongst people who validly claim ages even 10 years younger has never been observed by the New England Supercentenarian Study, which has enrolled 115 people aged 110 years or older as of December, 2010 (permission: The Daily Mail).

Some individuals, either purposefully or by mistake, claim that they are the oldest person in the world to bring notoriety to themselves and/or their family. They might do so completely convinced of their age, though they are mistaken because they have either been told by others what their (revised) age is, or because of cognitive frailty, they have forgotten about an intentional or erroneous change in their birth date from a long time ago. For example, Mariam Amash was surrounded by her family during all the media attention paid to her while recently claiming to be 120 years old (Figure 5). In a February, 2008 Daily Mail article, Moshe Hazut, a local official in the Northern Israeli town where Ms. Amash lived, stated that a birth certificate did not exist: “The woman was born during the Ottoman period, a time when the population registry was very inaccurate” [73]. Also, the age of her youngest son, Mohamed, 54 years old, would indicate that she gave birth at the age of 66, which particularly before the advent of modern fertility treatments, would be unheard of.

### 3.9. Military Age Misreporting

Motivations regarding military status can lead to age misreporting. In some cases, this is to make a child old enough to serve, in others to avoid war service. Various people in the 1940s and 1950s falsely claimed to be Confederate veterans, (and thus born in the mid-1800s) invoking a myth of Southern longevity. Arguing for the “Lost Cause,” it was even stated that “if we cannot beat ‘em, we can outlive ‘em”. Not one of the claimed Confederate ages turned out to be correct, and most were not even veterans [74]. For example, John Salling claimed to be 112 (http://generaljohnsalling.com/), but was 101. Walter Williams, the “last Confederate veteran” was not 117, but 105 years old (and not a veteran, either). Williams' motivation for age inflation could be partly monetary. He apparently inflated his age only in 1934, when a Confederate pension was offered during the Depression in Texas. At the time, Confederate promoters also claimed him as a heritage symbol [74].

Also of note, the last Union veteran, Albert Woolson, claimed to be 109 years old but research has shown that he was just 106 according to the census [74]. The oldest Union veteran, James Hard, claimed to be 111 years old in 1953 but investigation showed him to be 109 [74]. Fictionalized accounts of extreme age and war service continue to the present day. Merlyn Krueger recently claimed to be born in 1895 as well as a World War I veteran, but research by R.D. Young has shown him to be born in 1917. In some cases, the age is off by just a few years: Frank Buckles, the last surviving U.S. veteran of WWI, claimed to be “21” in 1917 so he could join the army, but he was just 16 years old then (and thus aged 110, but “115” according to his recruitment papers) [75].

In addition to the late-life military age myths, some men overstated their age earlier in an effort to avoid military service: claiming to be too old to be in the draft. If a man was 40 but claimed to be 50 in World War II, he could avoid military service, but would have to maintain the claim afterwards. This was common in Eastern Europe during World War II, when the draft age was often as high as 45. The claim that Pawel Parniak was 116 when he died in 2006 was just such an example. Research by R.D. Young showed that his mother was born in 1875 (just a 14-year generation gap) and that Mr. Parniak attempted to avoid recruitment in World War II (he was drafted anyway, despite being “49” on paper in 1939). It is far more likely that Mr. Parniak was closer to 111, having added about five years to his age in an attempt to avoid military service (it should be noted that he was also a World War I veteran), and his mother more likely gave birth to him at age 19 than 14.

### 3.10. Administrative Registration Errors

Administrative errors are an important source of inaccurate age claims, especially in more developed countries. For example, Damiana Sette in Sardinia was falsely noted to have died at the age of 110 years. In fact, she died at 107 years. The error was made several decades earlier in the transcription of administrative data. Damiana's data was replaced by those of her older sister who died at the age of 2 years, a few months before Damiana was born [34]. In Belgium, according to Michel Poulain's experience, the proportion of false claims due to administrative errors increases with age starting at 1% at age 100, 5% at 105, 50% at 110, and 100% at age 115. The reasons for such false cases include persons who emigrated abroad without reporting their birth date and accidentally unreported deaths (as opposed to purposefully unreported deaths, as in category 11, below) that result in administrative survivors. 

Errors in the recorded birth date (generally a foreign-born person with inappropriate documentation for date of birth) are an important cause. For example, Kamato Hongo of Japan was under the impression that she was 116 years old in 2003, thus making her the oldest person in the world at the time. However, Michel Poulain's research showed that a likely administrative error in her date of birth casts doubt upon the claim [34]. Eva Jourdan of France was noted to be “112,” but subsequent investigation discovered that she died at 102 and someone had copied “1890” as “1880” on a document (personal communication with INSERM, France). This suggests that original documentation is more reliable than copies of documentation. Whenever original documents can be secured, they are preferable to copies.

### 3.11. Unreported Deaths (Pension or Social Entitlement Fraud)

Pension fraud claims have proven to be a major contributor to extreme age claims. In some cases, relatives with a similar name have continued to fraudulently collect a pension. For example, Pearl Hackney claimed to be 117 years old but was later noted to be 93. In this case, she assumed the identity of an aunt with the same name (this claim was investigated by the GRG, Jeff Knight). Others likely claimed an older age during middle age for the likely purpose of prematurely collecting social security. Eddlee Bankhead of Pennsylvania changed his age from 57 to 73 when he applied for social security in 1956, adding 16 years to his age. He died at the claimed age of “116”, but census research showed that he was actually born in 1899 (per investigation by R.D Young).

In Japan, families have been caught collecting pensions for relatives that disappeared 40+ years ago (but were still listed as living, on paper, aged 110+) or even keeping the dead body of a relative in a room. One man, supposed to be 107, had been dead for more than a decade as the family collected money and gifts [76]. In 2010, a similar scandal erupted as Japanese officials launched an investigation into at least 200 suspected cases of fraudulent pension claims involving people claimed to be very old but who likely died many years ago, including a person who would have been 125 years old if still living [77]. “111”-year-old Sogen Kato, turned out to have been dead since 1978, since the age of 79 [76]. A similar form of pension fraud recently took place in Greece, where 300 of 500 supposed living centenarians were found to have died in the previous seven years [78]. This is a reminder that the three minimum requirements for age validation of supercentenarians include proof of survival to an age of 110+ years; proof of birth alone is not sufficient.

## 4. Discussion

Extraordinary claims of extreme longevity regularly surface in the media without circumspection. These claims are often times not benign, however, given underlying motivations. In our experience, the vast majority of claims over the age of 110 and nearly all of those over 115 years are false, and therefore such claims must be regarded with great care and scrutiny. 

The reporting of invalid ages as real can also cast a sensationalist shadow on the academic and responsible research of true supercentenarians. Extreme age claims do not deserve the benefit of the doubt, and without substantiating proof, like the Loch Ness monster and Bigfoot, they should be regarded as false.

Improvement in the quality of basic demographic data and the care with which it is managed may yield more reliable information as time moves forward, which will greatly enhance the ability to prove and disprove extreme age claims. But given human nature, however, and a number of the modern and historic sources of age misreporting that we list above, age validation will continue to need to be an integral part of valid exceptional longevity research. Even areas thought to have complete birth registration have seen problems with immigrant cases, unreported deaths, pension fraud, and the like. The older the alleged age of a longevity claim, the more in-depth must be the validation procedure.

Furthermore, as long as outrageous claims continue to be reported in the press without even a note of skepticism, they lend support to futurists and quacks who make claims that the average person today has the opportunity to achieve these purported ages. It is our hope that a more general knowledge of the typical circumstances under which age misreporting occurs may be helpful in decreasing irresponsible coverage of such claims and underscores the importance of skepticism and taking the substantial effort to proving or disproving a claim of extreme longevity.

## Figures and Tables

**Table 1:** Social Security Death Index-generated frequencies of alleged supercentenarians whose deaths were reported between 1980 and 2009 and validation rate.

Age range	Total claims	Number validated	Validation rate (%)
109-110	1106	183	16.6%
110-111	610	219	35.9%
111-112	372	109	29.3%
112-113	257	63	24.5%
113-114	190	27	14.2%
114-115	222	16	7.2%
115-116	53	3	5.7%
116-117	39	0	0.0%
117-118	39	1	2.6%
118-119	32	1	3.1%
119-120	25	0	0.0%
120-121	7	0	0.0%
121-122	18	0	0.0%
122-123	9	0	0.0%
123-124	13	0	0.0%
124-125	8	0	0.0%
125-126	7	0	0.0%
126-127	6	0	0.0%
127-128	6	0	0.0%
128-129	3	0	0.0%
129-130	7	0	0.0%

Totals	3029	622	20.5%

Excluding 109-110:	1923	439	22.83%

Social Security Death Index-generated frequencies of alleged supercentenarians whose deaths were reported between 1980 and 2009. The number of these purported supercentenarians was compared with an age-validated list of supercentenarians, also who died during the same time period, generated by the Gerontology Research Group, to obtain the validation rate. The rate for the age 109-110 range was lower than might otherwise be expected because persons who died at age 109 (e.g., born in April 1870 and died in January, 1980) were counted as “not validated.”
